# Activation of Phosphatidylinositol-Linked Dopamine Receptors Induces a Facilitation of Glutamate-Mediated Synaptic Transmission in the Lateral Entorhinal Cortex

**DOI:** 10.1371/journal.pone.0131948

**Published:** 2015-07-02

**Authors:** Iulia Glovaci, C. Andrew Chapman

**Affiliations:** Center for Studies in Behavioral Neurobiology, Department of Psychology, Concordia University, Montréal, Québec, Canada; Karolinska Inst, SWEDEN

## Abstract

The lateral entorhinal cortex receives strong inputs from midbrain dopamine neurons that can modulate its sensory and mnemonic function. We have previously demonstrated that 1 µM dopamine facilitates synaptic transmission in layer II entorhinal cortex cells via activation of D_1_-like receptors, increased cAMP-PKA activity, and a resulting enhancement of AMPA-receptor mediated currents. The present study assessed the contribution of phosphatidylinositol (PI)-linked D_1 _receptors to the dopaminergic facilitation of transmission in layer II of the rat entorhinal cortex, and the involvement of phospholipase C activity and release of calcium from internal stores. Whole-cell patch-clamp recordings of glutamate-mediated evoked excitatory postsynaptic currents were obtained from pyramidal and fan cells. Activation of D_1_-like receptors using SKF38393, SKF83959, or 1 µM dopamine induced a reversible facilitation of EPSCs which was abolished by loading cells with either the phospholipase C inhibitor U-73122 or the Ca^2+^ chelator BAPTA. Neither the L-type voltage-gated Ca^2+^ channel blocker nifedipine, nor the L/N-type channel blocker cilnidipine, blocked the facilitation of synaptic currents. However, the facilitation was blocked by blocking Ca^2+^ release from internal stores via inositol 1,4,5-trisphosphate (InsP_3_) receptors or ryanodine receptors. Follow-up studies demonstrated that inhibiting CaMKII activity with KN-93 failed to block the facilitation, but that application of the protein kinase C inhibitor PKC(19-36) completely blocked the dopamine-induced facilitation. Overall, in addition to our previous report indicating a role for the cAMP-PKA pathway in dopamine-induced facilitation of synaptic transmission, we demonstrate here that the dopaminergic facilitation of synaptic responses in layer II entorhinal neurons also relies on a signaling cascade dependent on PI-linked D_1_ receptors, PLC, release of Ca^2+^ from internal stores, and PKC activation which is likely dependent upon both DAG and enhanced intracellular Ca^2+^. These signaling pathways may collaborate to enhance sensory and mnemonic function in the entorhinal cortex during tonic release of dopamine.

## Introduction

The entorhinal cortex is an essential parahippocampal region through which multimodal sensory information from the neocortex reaches the hippocampal formation [1], and it is thought to contribute significantly to the sensory and mnemonic functions of the medial temporal lobe [2]. Functionally, the medial entorhinal cortex is linked to areas that are involved in spatial processing such as the subicular complex, postrhinal, and retrosplenial cortex [3, 4], whereas the lateral division is linked to object and odor recognition and familiarity, and receives strong inputs from the perirhinal cortex [5–9]. In addition, the cell islands of layer II of the lateral entorhinal cortex receive one of the largest cortical projections from midbrain dopamine neurons that contribute to appetitive motivation and learning [10–15].

Although the exact functions played by dopamine in the superficial layers of the lateral entorhinal cortex are presently poorly understood, effects of dopamine on synaptic transmission suggest that low concentrations of dopamine may act to enhance the salience of synaptic inputs received from sensory regions. The effects of dopamine on synaptic transmission in the entorhinal cortex are concentration-dependent, wherein high concentrations of dopamine (50–100 μM) result in a *suppression* of glutamate-mediated synaptic responses [16, 17] via a D_2_ receptor-dependent mechanism [18], and lower concentrations of dopamine (1–10 μM) induce a D_1_-receptor-mediated *facilitation* of synaptic responses [19, 20]. Functionally, low to moderate levels of D_1_-like receptor activation enhance synaptic transmission and working memory function in the prefrontal cortex [21–23], and dopamine may act similarly in the superficial layers of the lateral entorhinal cortex.

Our laboratory has recently investigated the intracellular signaling pathways mediating the dopaminergic facilitation of glutamatergic transmission in fan and stellate cells of layer II of the entorhinal cortex. Similar to findings in the prefrontal cortex and hippocampus [24, 25], we established that binding of dopamine to D_1_-like, but not D_2_-like, receptors leads to a rapid and reversible increase in the amplitude of glutamatergic excitatory postsynaptic currents (EPSCs) via a signaling cascade that is dependent on increased activation of the cyclic AMP-protein kinase A (PKA) pathway [20]. Increased PKA activity can increase the phosphorylation of the dopamine- and cAMP-regulated phosphoprotein, 32 kDa, (DARPP-32) and of inhibitor 1 (I-1), which are potent inhibitors of protein phosphatase 1 (PP1), a phosphatase that reduces synaptic responses by dephosphorylating AMPA receptors during basal conditions [26–28]. We also found that the dopaminergic facilitation of AMPA-mediated EPSCs was dependent upon PP1 activity [20]. These results provided the first evidence that PKA-mediated inhibition of PP1 contributes to the dopaminergic facilitation of AMPA-mediated synaptic responses.

In addition to the cAMP-PKA pathway, D_1_-like receptor activation leading to a facilitation of synaptic transmission has also been reported to depend on increases in postsynaptic calcium in the striatum [29, 30], prefrontal cortex [24, 31], and hippocampus [32]. Our findings are similar, wherein intracellular application of the calcium chelator BAPTA completely blocked the dopamine-induced facilitation of EPSCs in the entorhinal cortex. However, the intracellular cascade linking dopamine to increased intracellular calcium, and the mechanism through which calcium may contribute to the synaptic enhancement remained unclear.

The present experiments aimed to determine the origin of increased intracellular calcium and the signaling cascade required for dopamine-induced enhancement of AMPA-mediated synaptic transmission in layer II principal cells of the entorhinal cortex. Recordings in other areas have shown that D_1_ receptors linked to G_s_ proteins that stimulate cAMP-PKA can enhance calcium currents via PKA-mediated phosphorylation of both L- and N-type voltage-gated calcium channels (VGCCs) [33–35]. Because we found that PKA signaling was required for the dopamine-mediated facilitation of EPSCs [20], and PP1 also regulates phosphorylation of L- and N-type VGCCs [36–38], our first experiments tested the involvement of L- and N-type VGCCs in the dopaminergic facilitation of synaptic transmission. In addition to G_s_-linked D_1_ receptors, phosphatidylinositol (PI)-linked D_1_ receptors coupled to G_q_ proteins that stimulate PLC to increase production of diacylglycerol (DAG) and inositol triphosphate (InsP_3_) can lead to increased cytosolic calcium levels by binding to InsP_3_ receptors that mediate calcium release from internal stores [39–42]. We therefore also assessed the role of PI-linked D_1_ receptors, PLC, and release of Ca^2+^ from internal stores. Our results show a role for PI-linked D_1_ receptors in the facilitation of EPSCs that is dependent on InsP_3_-medited release from internal stores, and further indicate that the Ca^2+^-dependent kinase PKC is required for the dopaminergic enhancement of EPSCs in the entorhinal cortex.

## Materials and Methods

### Ethics Statement

All procedures outlined in this study were carried out in accordance with the guidelines of the Canadian Council on Animal Care, and the protocol was approved by the Concordia University Animal Research Ethics Committee (Permit Number: 30000253) [S1 ARRIVE Checklist]. All animals were deeply anesthetized with halothane prior to decapitation, and all efforts were made to minimize suffering.

### *In Vitro* Slice Preparation

Recordings were obtained from brain slices collected from 4–9 week-old male Long-Evans rats (Charles River). Brains were quickly extracted and submerged into an ice cold, high-sucrose artificial cerebrospinal fluid cutting solution (ACSF; saturated with 95% O_2_ and 5% CO_2_, pH ≈7.4) containing (in mM) 250 sucrose, 2 KCl, 1.25 NaH_2_PO_4_, 7 MgCl_2_, 26 NaHCO_3_, 0.5 CaCl_2_ and 10 dextrose. All drugs were obtained from Sigma-Aldrich unless indicated otherwise. Horizontal slices containing the entorhinal region were obtained using a vibratome (300 **μ**M thick; WPI, Vibroslice, Sarasota, USA). There was a recovery period of at least one hour in normal ACSF containing (in mM) 124 NaCl, 5 KCl, 1.25 NaH_2_PO_4_, 2 MgSO_4_, 2 CaCl_2_, 26 NaHCO_3_, and 10 dextrose (pH ≈7.4; 300–310 mOsm; ~22 ºC). During recordings, individual slices were submerged in ASCF (2 ml/min) and fixed using a nylon net, and were visualized using an upright microscope (Leica, DM-LFS) equipped with a 40x objective and differential interference contrast optics. Layer II of the lateral entorhinal cortex was distinguished from layers I and III based on the presence of clusters of cells [1].

### Stimulation and Recording

Recording pipettes used for whole-cell recordings were pulled from borosilicate glass (1.0 mm OD, 2.7 to 6 MΩ) and were filled with a solution containing (in mM) 140 K-gluconate, 5 NaCl, 2 MgCl_2_, 10 HEPES, 0.5 EGTA, 2 ATP-Tris, and 0.4 GTP-Tris (pH adjusted to 7.2–7.3 with KOH). The formation of a tight seal between the pipette and soma (1–4 GΩ) was obtained using gentle negative pressure, and a stronger pressure was applied to obtain whole-cell configuration. Neurons were allowed to stabilize for 10–15 minutes prior to recordings to allow for diffusion of intracellular drugs where applicable. Synaptic responses were evoked using a bipolar stimulating electrode made from two tungsten electrodes (~1 MΩ, FHC Inc.) placed in layer I of the lateral entorhinal cortex, approximately .1 to .2 mm rostral to the recording electrode. Synaptic responses were evoked by 0.1 ms-duration constant current pulses delivered using a stimulus timer and isolation unit (WPI, Models A300 and A360). Stimulus intensity was adjusted to evoke responses of roughly 70% of maximal without evoking action potentials (131 ± 15 **μ**A). Current and voltage clamp recordings were obtained using an Axopatch 200B amplifier, and were filtered at 5–10 kHz, then digitized at 20 kHz (Axon Instruments, Digidata 1322A).

Electrophysiological characteristics and firing properties of entorhinal neurons were characterized prior to synaptic recordings by recording membrane potential responses to 500 ms duration current pulses (range -200 to +60 pA). Responses were analyzed using the Clampfit 8.2 software package (Axon Instruments). Inward rectification was quantified as the ratio between peak and steady-state input resistances in response to -200 pA hyperpolarizing current pulses (rectification ratio). Spike properties were measured from the first action potential evoked in response to positive current injection. Action potential amplitude was calculated from resting membrane potential and action potential duration and afterhyperpolarization were measured from action potential threshold.

### Synaptic Currents

**Fig 1:**
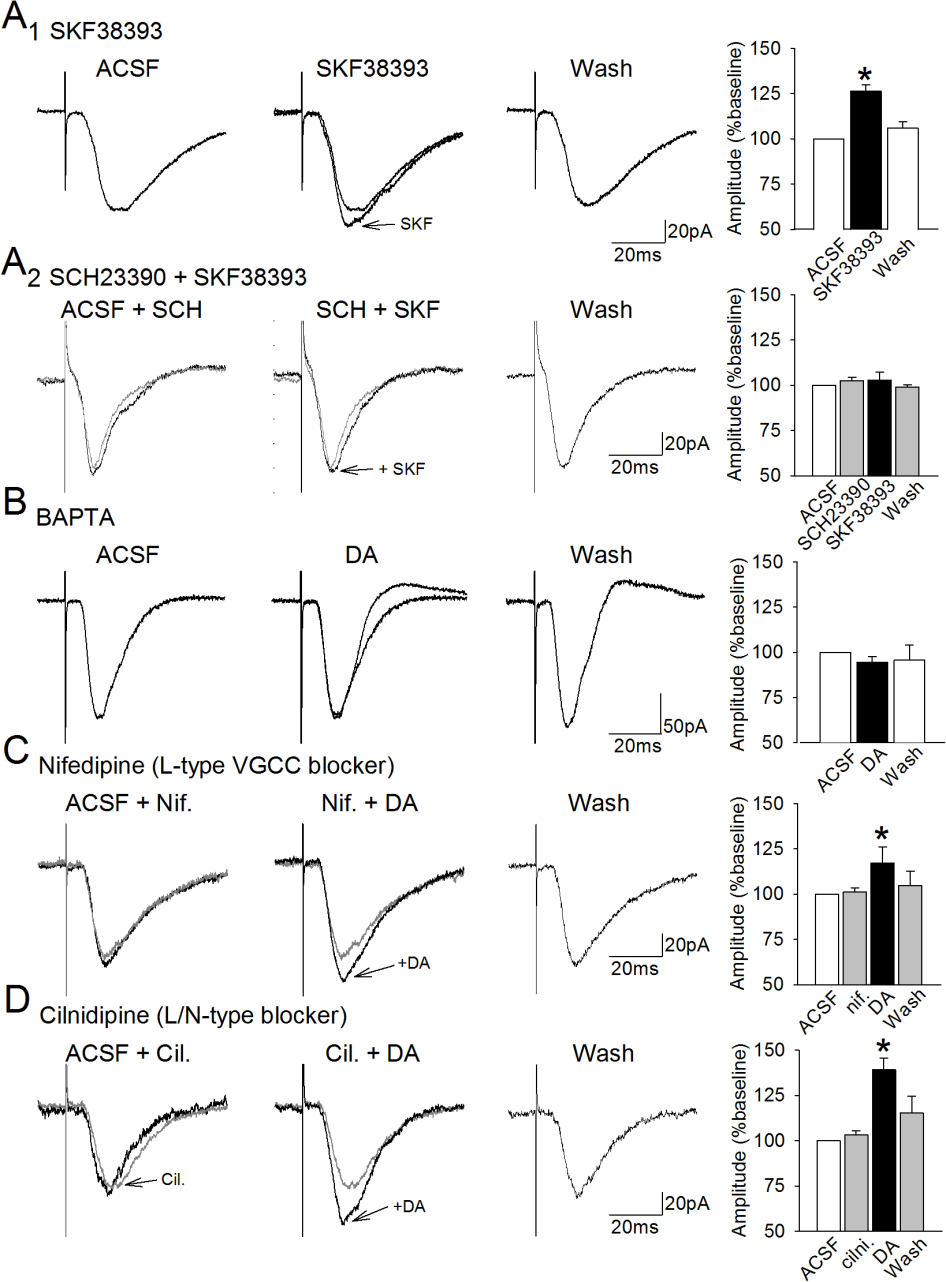
Dopaminergic facilitation of EPSCs is dependent on intracellular calcium, but not L- or N-type VGCCs. **A.** Bath application of the dopamine D_1_-receptor agonist SKF38393 (10 **μ**M) induces a reversible facilitation of the amplitudes of glutamate-mediated excitatory postsynaptic currents in layer II lateral entorhinal cortex neurons (A_1_). Traces show averaged EPSCs for a neuron before, during, and after 5-min application of SKF38893. The histogram at right shows mean EPSC amplitudes for the group of cells. Bars indicate ± one SEM and the asterisk indicates p < 0.05. In addition, the facilitation induced by SKF38393 is blocked in the presence of the D_1_ receptor blocker SCH23990 (A_2_). **B.** Amplitudes of EPSCs recorded from cells filled with the Ca^2+^ chelator BAPTA (10 **μ**M) remained stable during dopamine (DA) application. **C,D.** Bath-application of the L-type voltage-gated calcium channel (VGCC) blocker nifedipine (10 **μ**M; C) or of the L/N-type VGCC channel blocker cilnidipine (10**μ**M; D) failed to block the dopaminergic facilitation of EPSCs (*, p < 0.001). Traces for the baseline and VGCC blockers are superimposed.

**Fig 2:**
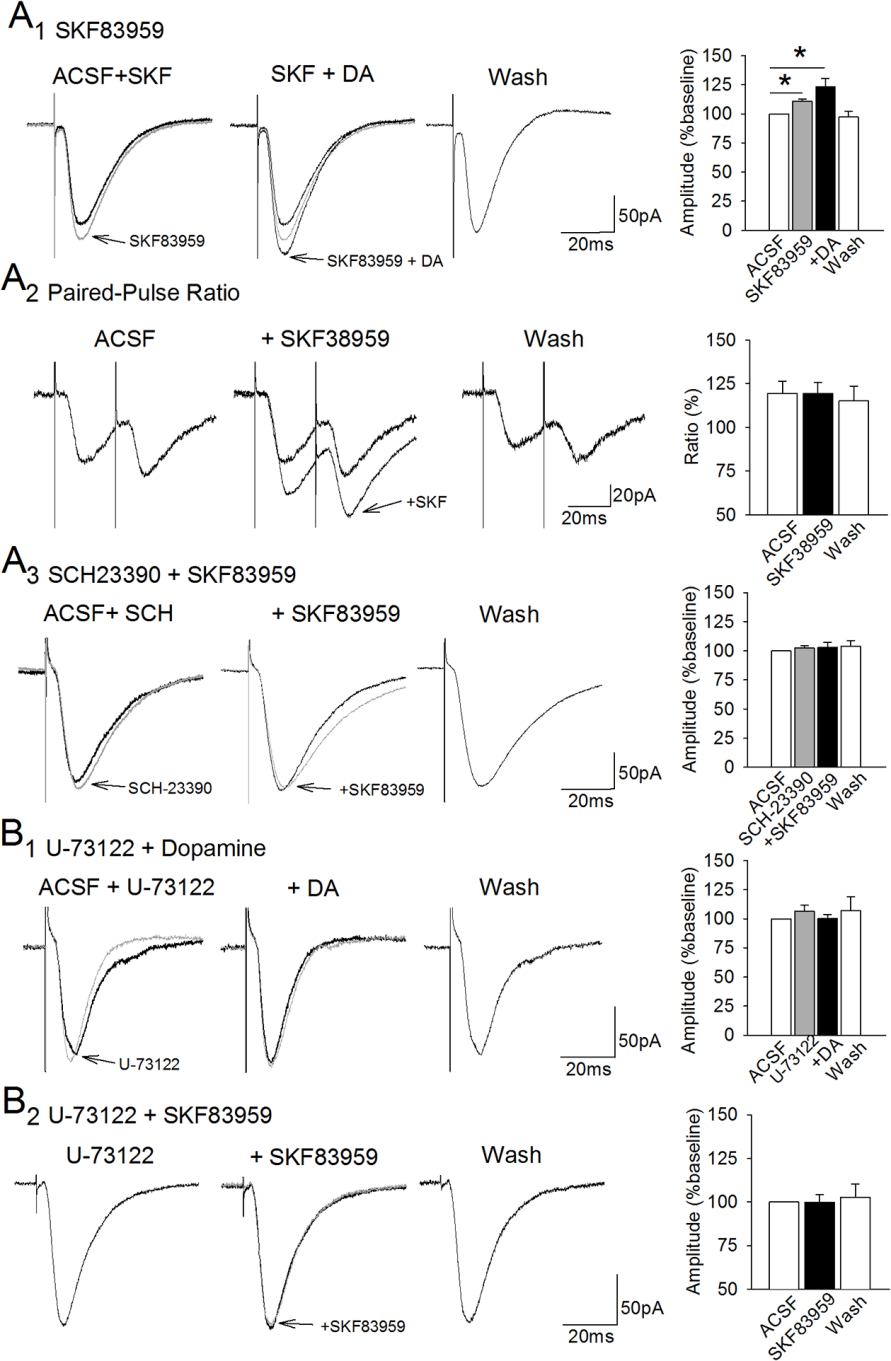
The facilitation of EPSCs induced by the D_1_ agonist SKF83959 is dependent upon PLC activation. **A.** Bath application of the selective PI-linked D_1_-receptor agonist SKF83959 (5 or 10 **μ**M) resulted in a significant enhancement of EPSCs (A_1_, superimposed traces at left; *, p < 0.05). Subsequent addition of dopamine was followed by a further non-significant increase in mean EPSC amplitude (middle traces; p = .06). The facilitation of EPSC amplitude by SKF38959 was not associated with a change in paired-pulse facilitation ratio (A_2_). Application of the D_1_ receptor antagonist SCH23390 (10 **μ**M; A_3_) blocked the facilitation induced by SKF83959. **B.** The PLC inhibitor U-73122 (10 **μ**M) had no effect on baseline EPSCs (B_1_, traces at left), but blocked the facilitation of EPSC amplitudes induced by dopamine (middle traces). Including U-73122 in the recording solution also blocked the facilitation of EPSC amplitudes induced by SKF83959 (B_2_).

The effects of dopamine receptor activation on evoked synaptic response in the entorhinal cortex were assessed by recording excitatory postsynaptic currents (EPSCs) at a holding potential of -60 mV before, during and after 5-min bath application of dopamine or dopamine receptor agonists. We previously found that early and late inhibitory synaptic potentials are not affected by dopamine application in layer II entorhinal neurons [20] and GABA receptor blockers were therefore not included in experiments. Cells occasionally showed outward currents at latencies following the peak of EPSCs during drug application or wash (e.g., Figs 1B and 2A _1_), but these were not associated with altered patterns of drug effects on EPSC amplitude. Ten to twenty EPSCs were evoked by single pulses delivered once every 15 sec during each recording condition. Input resistance, access resistance and capacitance were monitored via responses to -10 mV 50 ms voltage steps delivered before each evoked synaptic response, and recordings were discontinued if the values changed by >15%. The dopamine-induced facilitation of AMPA receptor-mediated transmission [20] was replicated by recording EPSCs at a holding potential of -60 mV during a baseline period, following 5-min application of the D_1_-like receptor agonists SKF38893 (10 **μ**M) or SKF83959 (5 or 10 **μ**M), and following a 20 min washout period in normal ACSF. Possible increases in glutamate release induced by the PI-linked dopamine receptor agonist SKF83959 (10 **μ**M) were assessed in some neurons by monitoring responses to pairs of stimulation pulses separated by 30 ms [20].

The role of intracellular calcium in the dopamine-induced facilitation of EPSCs was assessed by including the calcium chelator 1,2-bis(2-aminophenoxy)ethane-N,N,N′,N ′-tetracetic acid (BAPTA, 10 **μ**M) in the intracellular solution, and EPSCs were recoded prior to and during 5-min application of dopamine (1 **μ**M), and after 20 min of washout. The dependence of the dopaminergic facilitation on voltage-gated calcium channels was assessed by applying 1 **μ**M dopamine during constant bath application of either the selective L-type channel blocker cilnidipine (10 **μ**M), or the mixed L/N-type blocker nifedipende (10 **μ**M). There was a 10 min wash-out period in the presence of the blocker, and during an additional 10 min in normal ACSF. The contribution of InsP_3_-receptors and ryanodine receptors was assessed by including either heparin (1 mM) or dantrolene (20 **μ**M) in the recording solution, and by comparing EPSCs recorded before, during, and after dopamine. Other signaling molecules were tested using the bath application of the PLC inhibitor U-73122 (10 **μ**M), and intracellular application of the PKC inhibitor PKC(19–36) (1 **μ**M; Tocris Bioscience), or the calcium/calmodulin-dependent protein kinase II (CaMKII) blocker KN-93 (5 **μ**M, dissolved in dimethylsulfoxide (DMSO) with a final concentration of 0.1%; Tocris Bioscience). All recording solutions were stored at –20°C.

Changes in synaptic responses were analyzed using Clampfit 8.2 software (Axon Instruments). For each cell, at least 10 consecutive synaptic responses free from artifacts or action potentials were averaged for each phase of recordings, and the amplitudes of averaged responses were measured relative to the pre-stimulus baseline. Raw data were analyzed using modified Bonferroni planned comparisons and an alpha level of .05 [43] to assess changes in cellular properties and synaptic responses prior to and after drug application, as well as between baseline and washout to assess the reversibility of effects and stability of recordings. The data met the requirements for normality (Lilliefors' corrected Kolmogorov-Smirnov test), and are presented here as group means ± one standard error of the mean. Cohen’s d was calculated as a measure of effect size.

## Results

Effects of dopamine on evoked EPSCs were assessed in recordings from 97 layer II lateral entorhinal cortex neurons. Neurons were classified by their electrophysiological profile in current clamp recordings [44] as either fan cells that are characterized by a marked sag in the voltage response to strong hyperpolarizing current injection (n = 45; rectification ratio: 1.23 ± 0.09) or pyramidal neurons that fire regularly with no substantial sag response (n = 52; rectification ratio: 1.01 ± 0.01). Drug effects on synaptic responses were not dependent on cell type, and the data reported here reflect mean responses in mixed groups of neurons.

### The facilitation of responses induced by D_1_-receptor activation is calcium-dependent

Consistent with our previous findings that 1 **μ**M dopamine induces a reversible D_1_-like receptor-dependent facilitation of AMPA receptor-mediated EPSCs in entorhinal cells [20], we found here that 5 min bath application of the selective D_1_-receptor agonist SKF38393 (10 **μ**M) facilitates EPSCs in lateral entorhinal cortex cells held at -60 mV (Fig 1A_1_). Responses were increased by SKF38393 to 126.3 ± 3.5% of baseline responses (t = 2.63, p = 0.04, n = 6, d = 1.3). The facilitation was reversible, and responses recorded after 20 min washout did not differ significantly from baseline (105.9 ± 3.7%). The facilitation of synaptic responses was not associated with changes in cellular input resistance (89 ± 7 vs. 87 ± 7 MΩ; t = 0.98, p = 0.36) or capacitance (169 ± 31 vs. 184 ± 30 pF; t = 1.32, p = 0.24). The facilitation induced by SKF383934 was also blocked by the D_1_ receptor antagonist SCH23990 (10 **μ**M; 102.7 ± 4.4% of baseline responses; t = 0.11, p = 0. 92, n = 6), indicating that it is dependent upon activation of D_1_ dopamine receptors (Fig 1A_2_), consistent with our previous findings that the dopaminergic facilitation of synaptic responses is dependent upon activation of D_1_ receptors [20].

Increases in intracellular calcium contribute to the D_1_ receptor-dependent enhancement of glutamatergic responses in other regions [24, 45, 46]. We therefore assessed the role of intracellular Ca^2+^ in the facilitation of EPSCs by including the Ca^2+^ chelator BAPTA (10 **μ**M) in the intracellular solution. Comparisons of action potential waveforms recorded immediately, and 10 to 15 min after break-in in three of the cells filled with BAPTA showed an increase in action potential duration (5.6 ms to 8.2 ms) and a reduction in medium afterhyperpolarization (2.9 mV to 1.5 mV), consistent with reduced intracellular Ca^2+^. The dopaminergic facilitation of EPSC amplitudes was blocked by BAPTA (94.6 ± 3.1% of baseline; n = 7, t = 0.51, p = 0.63; Fig 1B) indicating that increases in intracellular calcium are required for the facilitation effect.

Dopamine is known to enhance calcium influx via PKA-mediated phosphorylation of L-type voltage-gated calcium channels in other neurons [30, 45, 46], and we have shown that facilitation of AMPA-receptor currents in entorhinal neurons is dependent on increased PKA activity [20]. However, we found here that bath-application of the selective L-type voltage-gated calcium channel blocker nifedipine (10 **μ**M) did not block the dopaminergic facilitation of EPSCs (n = 8; 118.7 ± 8.5% of baseline, t = 2.67, p = 0.03, d = .41), indicating that calcium influx through L-type VGCCs is not required for the facilitation of EPSCs. The facilitation induced by dopamine reversed during washout in nifedipine (see Fig 1C), and remained at baseline values during 10 min subsequent washout in ACSF (105.8 ± 7.4% of baseline, t = 0.33, p = 0.76).

N-type voltage-gated calcium channels are also present in the entorhinal cortex [47], and calcium influx via N-type channels could also be enhanced by D_1_-like receptor activation and PKA-mediated phosphorylation [30, 48]. Bath-application of the combined L/N type VGCC blocker cilnidipine (10 **μ**M) alone did not change the amplitude of EPSCs (n = 6; 103.0 ± 2.5% of baseline, t = 0.16, p = 0.9), but subsequent co-application of dopamine increased EPSCs in these cells to 139.3 ± 6.5% of baseline (t = 8.56; p = 0.0004, d = 1.07), and responses returned to baseline during 20 min washout in ACSF (110.3 ± 5.4% of baseline, t = 0.55, p = 0.6; Fig 1D). In addition, the dopaminergic facilitation of EPSCs during application of nifedipine and cilinidipine was not associated with a change in holding current or input resistance. Thus, neither L- nor N-type VGCCs are required for the dopaminergic facilitation of EPSCs in entorhinal neurons.

### The facilitation is dependent upon phosphatidylinositol-linked dopamine receptors and internal calcium stores

Because voltage-gated calcium channels were not required, we then investigated the role of phosphatidylinositol (PI)-linked D_1_-like receptors that lead to activation of PLC and production of InsP_3_ that could result in release of calcium from internal stores [41, 49, 50]. We tested the involvement of this receptor using SKF83959 which is the most selective agonist available for PI-linked D_1_-dopamine receptors [51–53, 40, 41]. The amplitude of EPSCs increased during application of 5–10 **μ**M SKF83959 to 110.9 ± 1.9% of baseline values (n = 6; t = 4.06, p = 0.009, d = .44), indicating that these receptors could be involved in the synaptic effects of dopamine (Fig 2A_1_). Subsequent co-application of 1 **μ**M dopamine was associated with a further, but statistically non-significant, increase in EPSC amplitudes to 123.2 ± 7.3% of baseline values (n = 7; t = 2.18, p = 0.06, d = .35) that is consistent with the added involvement of cAMP-PKA-linked D_1_-like receptors described previously [20]. The effects were reversible, and EPSC amplitudes returned to baseline values during a 20 min washout period in ACSF (97.3 ± 5.0% of baseline, n = 3; t = 2.68, p = 0.12; Fig 2A_1_). The facilitation of synaptic responses was not associated with changes in resting membrane potential (-55.7 ± 2.6 mV vs -54.3 ± 3.1 mV; t = -2.35, p = 0.07), holding current (-4.6 ± 16.5 vs -7.3 ± 17.6 pA; t = 1.05, p = 0.33), or membrane resistance (115.8 ± 34.7 vs 110.2 ± 34.3 MΩ; t = 1.95, p = 0.12). Because activation of presynaptic PI-linked D_1_ receptors can lead to increases in glutamate [54], we also assessed paired-pulse facilitation ratios before and after application of SKF83959 in a separate group of neurons. The significant facilitation of responses (119.2 ± 8.5% of baseline values, n = 6; t = 2.74, p = 0.04, d = 0.32) was not associated with significant alteration in paired-pulse ratio, however (Fig 2A_2_; 119.5 ± 6.9 vs. 119.6 ± 6.2, t = 0.095, p = 0.93) suggesting that changes in presynaptic release do not contribute to the effect. We also found that the facilitation induced by SKF83959 (5 **μ**M) was blocked in the presence of the D_1_ receptor antagonist SCH23990 (10 **μ**M; 101.8 ± 3.9% of baseline responses; t = 0.19, p = 0. 86, n = 6), indicating that it is dependent on D_1_ receptors (Fig 2A_3_).

Because PI-linked D_1_ receptors stimulate phospholipase C [55, 56], we tested whether or not PLC is required for the dopaminergic facilitation of EPSCs using bath-application of the PLC inhibitor U-73122 (10 **μ**M). Responses remained stable following application of U-73122 (n = 8; 106.6 ± 5.4% of baseline, t = 0.11, p = 0.91), and the presence of U-73122 also blocked the dopamine-induced enhancement of EPSCs (100.5 ± 3.3%, t = 0.03, p = 0.98; 107.1 ± 11.9% of baseline during washout, t = 0.01, p = 0.70; Fig 2B_1_) indicating that the dopaminergic facilitation requires activation of PLC. Intracellular application of U-73122 (10 **μ**M) also prevented facilitation of EPSCs induced by the putative PI-linked D_1_-receptor agonist SKF83959. The responses remained stable following application of SKF83959 (5 **μ**M; n = 7; 99.9 ± 4.3% of baseline, t = 0.52, p = 0.62; Fig 2B_2_), as well as during washout (102.8 ± 7.6% of baseline, t = 0.005, p = 0.99) indicating that the facilitation induced by SKF83959 is dependent on activation of PLC.

**Fig 3:**
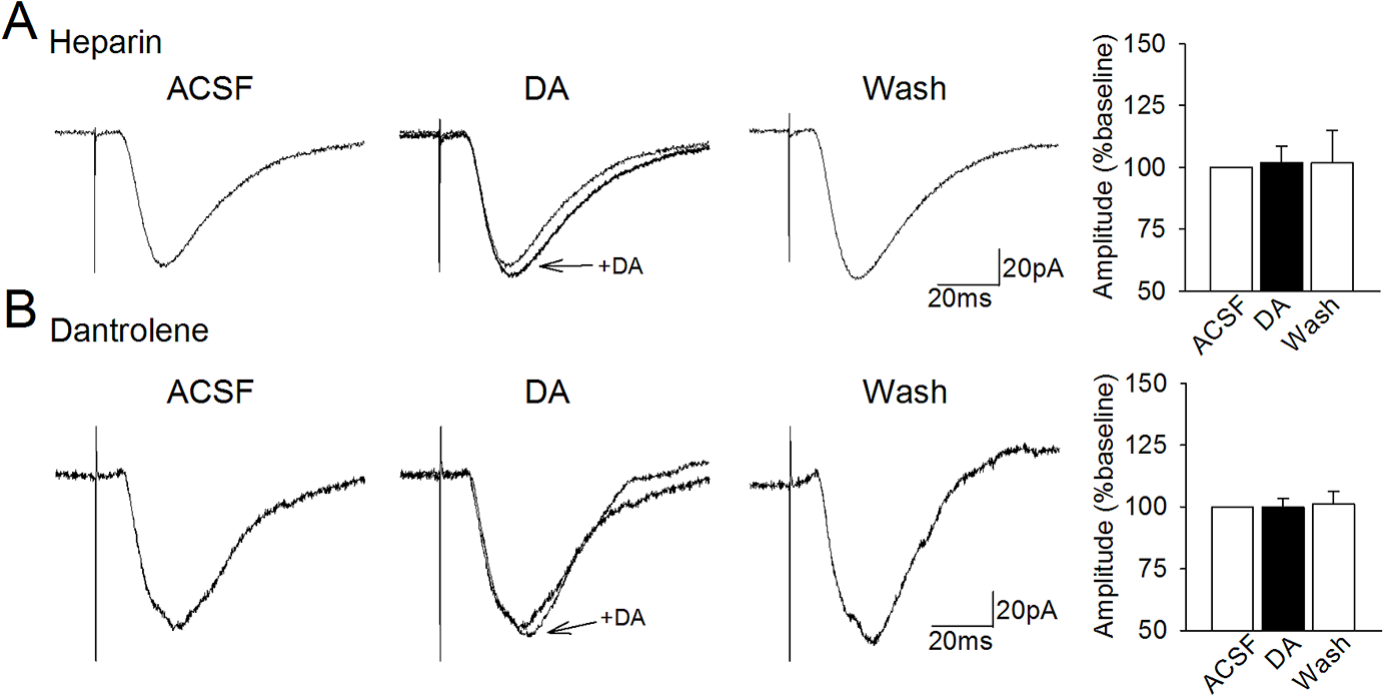
The DA-induced facilitation is dependent upon activation of both InsP_3_ and ryanodine receptors. **A.** Including the InsP_3_-receptor blocker heparin (1 mM) in the intracellular recording solution blocked the dopaminergic facilitation of EPSCs. **B.** Including the ryanodine receptor blocker dantrolene (20 **μ**M) in the recording solution also blocked the facilitation of EPSCs by dopamine.

PLC-induced production of InsP_3_ leads to increased cytosolic Ca^2+^ via binding to InsP_3_ receptors and the resulting release of Ca^2+^ from internal stores [56]. To test whether InsP_3_ receptors might mediate increases in Ca^2+^ required for the dopaminergic facilitation of EPSCs, we included the InsP_3_R blocker heparin (5 **μ**M) in the intracellular recording solution. Bath application of dopamine had no significant effect on EPSC amplitudes in heparin-filled neurons (97.6. ± 8.1% of baseline responses, n = 8; t = 0.86, p = 0.45; Fig 3A) indicating that activation of InsP_3_ receptors is required for the facilitation effect.

Calcium release from internal stores is also mediated by ryanodine receptors (RyR), which are themselves activated by low to moderate increases in cytosolic Ca^2+^ [56]. In order to assess the role of Ca^2+^ release via RyR, we included the RyR blocker dantrolene (20 **μ**M) in the intracellular solution. Application of dopamine had no effect on EPSC amplitudes in cells filled with dantrolene (99.8 ± 3.7% of baseline, n = 7; t = 0.20, p = 0.85; Fig 3B) indicating that the dopamine-induced facilitation of EPSCs is dependent upon activation of RyR. The requirement for both InsP_3_-R and RyR activation suggests that InsP_3_ receptor activation followed by calcium-induced calcium-release via RyR may be required for the facilitation effect.

### Calcium-Dependent Kinases

**Fig 4:**
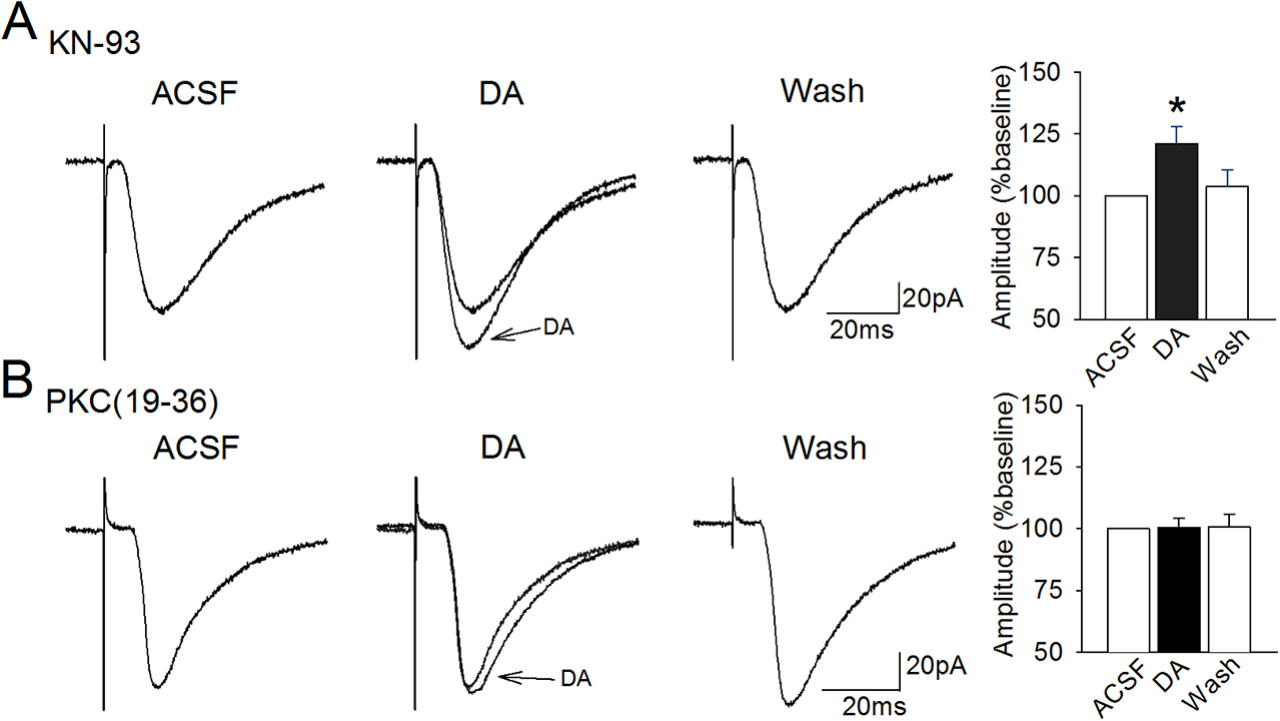
The dopaminergic facilitation is dependent on PKC but not on CaMKII. **A.** The Ca^2+^/calmodulin-dependent protein kinase (CaMKII) can enhance AMPA receptor function but we found that intracellular application of the CaMKII inhibitor KN-93 (5 µM) did not prevent the dopamine-induced facilitation of EPSCs (*, p < 0.05). **B.** In contrast, intracellular application of the PKC inhibitor PKC(19–36) reliably blocked the dopaminergic facilitation of EPSCs.

Increases in cytosolic calcium can enhance protein kinase activity in neurons, and protein kinases can rapidly regulate AMPA-receptor channel kinetics via receptor subunit phosphorylation [57–60]. In the hippocampus, dopamine D_1_-like receptor activation can lead to activation of calmodulin and subsequent activation of the Ca^2+^ and calmodulin-dependent protein kinase II (CaMKII) which may enhance AMPA single channel conductance via phosphorylation of the GluR1 subunit at ser^831^ [59–61]. Further, blocking CaMKII activity prevents the transient D_1_-mediated enhancement of EPSCs in prefrontal neurons [24]. However, we found that blocking CaMKII activity in layer II entorhinal cortex neurons using intracellular application of 5 **μ**M KN-93 did not block the dopamine-induced facilitation of EPSCs (n = 8; 120.8 ± 7.2% of baseline; t = 2.84, p = 0.025, d = 0.74; washout: 103.7 ± 6.8% of baseline; t = 0.64, p = 0.54; Fig 4A), indicating that activation of CaMKII is not required for the transient facilitation of synaptic transmission observed here.

Several studies also indicate that protein kinase C, which is activated by PLC-DAG and by Ca^2+^, can potentiate AMPA receptor-mediated currents in hippocampal neurons [61, 62]. In order to determine if increased Ca^2+^ might enhance EPSCs via increased PKC activity, we included the PKC inhibitor PKC(19–36) in the intracellular recording solution. The dopaminergic faciltiation of synaptic responses was blocked by PKC(19–36) (100.5 ± 3.6% of baseline during dopamine application, n = 8; t = 0.55, p = 0.6), and responses also remained stable during washout (100.6 ± 5.1%, t = 1.06, p = 0.33; Fig 4B). Therefore, increases in EPSCs amplitudes induced by dopamine are dependent upon activation of PKC that may lead to phosphorylation of AMPA receptors.

## Discussion

The lateral entorhinal cortex processes multimodal sensory information and has been linked to object recognition, olfaction, and mnemonic processes [2, 5–9]. As the lateral entorhinal cortex is one of the four major cortical targets of midbrain dopamine neurons [63], it is likely that dopamine availability in this area promotes the processing of reward-relevant stimuli [64]. Given that a low concentration of dopamine facilitates synaptic transmission onto principal cells of the lateral entorhinal cortex [18, 20], low, tonic levels of dopamine may enhance the salience of sensory inputs and promote memory formation during exploratory behavior in awake animals. However the intracellular pathways through which dopamine modulates glutamatergic transmission in layer II lateral entorhinal cortex neurons have hitherto remained unclear.

**Fig 5:**
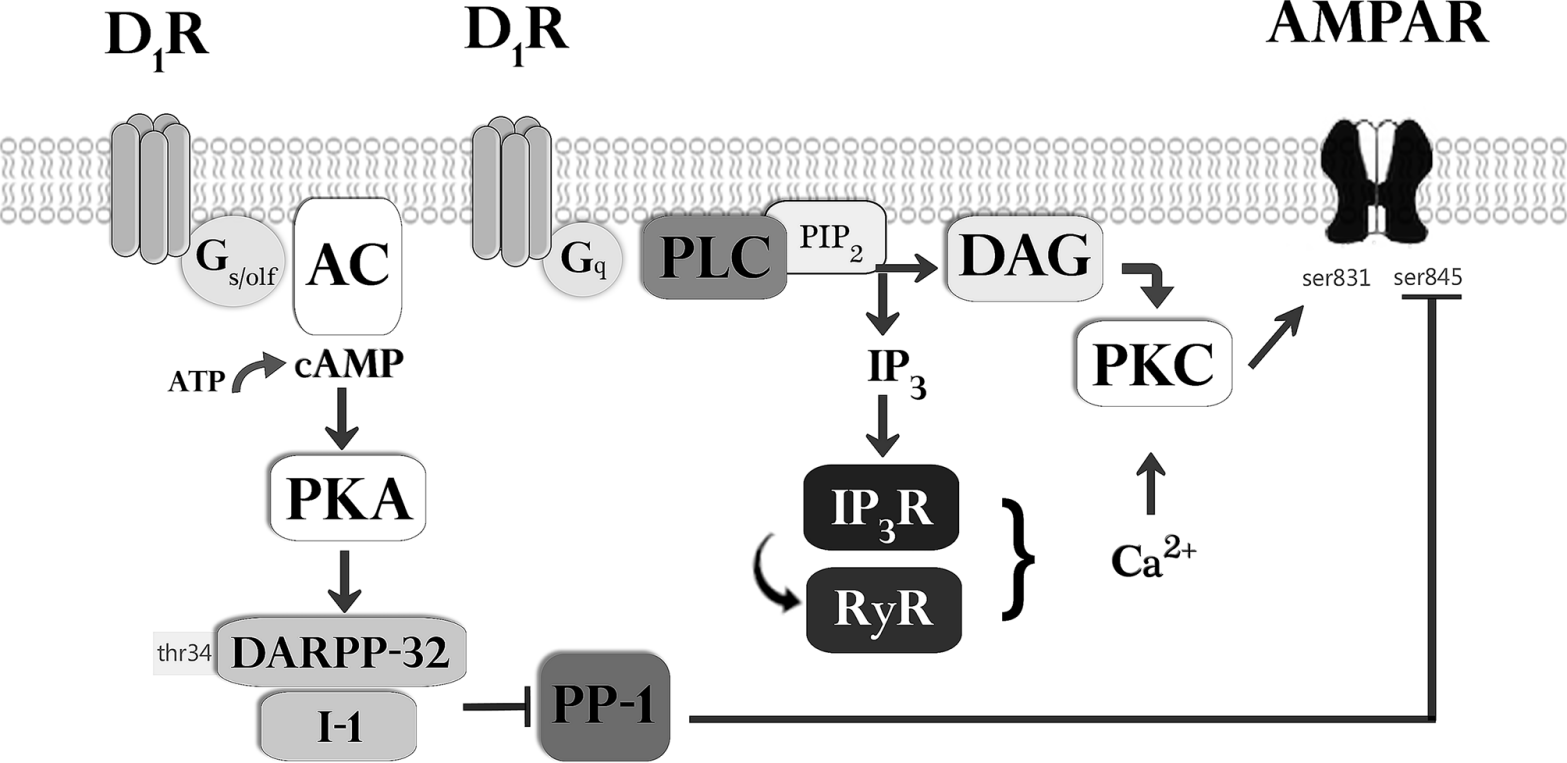
Proposed signaling pathways that govern the dopaminergic facilitation of AMPA-mediated EPSCs. Classical D_1_-receptors are coupled to adenylyl cyclase (AC) via G_s/olf_ proteins, and we have shown previously that the dopamine-induced facilitation is dependent upon the activity of both protein kinase A (PKA) and protein phosphatase 1 (PP1) [20]. The PKA-mediated activation of inhibitor 1 (I-1) or of DARPP32 (dopamine and cyclic AMP regulated phosphoprotein 32) at thr^34^ may inhibit activity of protein phosphatase 1 (PP1) and thereby potentiate EPSCs by reducing the dephosphorylation of AMPA GluR1 receptor subunits at ser^845^. The present results indicate that activation of D_1_-receptors coupled to phospholipase C (PLC) via G_q_ proteins is also required for the facilitation of EPSCs. PLC leads to production of diacylglycerol (DAG) and InsP_3_ from PIP_2_. InsP_3_ triggers Ca^2+^ release from internal stores via InsP_3_ receptor (InsP_3_R) activation, and may also trigger Ca^2+^ induced Ca^2+^ release via ryanodine receptors (RyR). Both increased cytosolic Ca^2+^ and DAG can activate protein kinase C (PKC) that can enhance AMPA receptor function via phosphorylation at ser^831^.

It is well-acknowledged that activation of D_1_-like receptors linked to G_s/olf_ proteins can lead to increased glutamatergic transmission via increased activity in the cAMP-PKA pathway in other brain regions [24, 65] and we previously reported a similar PKA-dependent dopaminergic potentiation of AMPA currents in lateral entorhinal cortex slices [20]. In the present paper, we describe an additional intracellular pathway that depends on activation of PI-linked D_1_ receptors that are coupled to G_q_ proteins, which lead to increases in PLC activity, InsP_3_-dependent release of calcium from internal stores, and a PKC-dependent facilitation of glutamate-mediated synaptic responses (Fig 5). Full potentiation of glutamate transmission appears to require parallel activation of both PLC-dependent and PKA-dependent pathways, because activation of PI-linked D_1_-receptors alone produces a partial facilitation effect (Fig 2A1), and blocking signaling steps within either the PKA- or PLC-dependent signaling pathways blocks the full facilitation effect [20]. Although the role of SKF83959 as a selective PI-linked D_1_ receptor agonist has been recently questioned [66], our current findings demonstrate that the dopaminergic facilitation is dependent upon D_1_-receptors, PLC activity, Ca^2+^ signaling and PKC activity. These data point strongly to a role for PI-linked dopamine receptors and subsequent activation of the D1-PLC-Ca^2+^ signaling cascade. The involvement of both PKA- and PLC-dependent signaling cascades provides increased means through which other neuromodulators may gate synaptic transmission in lateral entorhinal cortex neurons, to either promote or restrict synaptic transmission.

The experiments presented here indicate the blockade of postsynaptic calcium or PKC activity blocks the facilitation effect of dopamine, indicating an involvement of postsynaptic signals, but it is also possible that activation of PI-linked D_1_ receptors on presynaptic terminals could contribute to the facilitation effect by enhancing glutamate release [54, 67]. However, similar to our previous findings for the application of dopamine [20], we found here that the facilitation of synaptic responses induced by the PI-linked D_1_ receptor agonist SKF83959 was not associated with a change in paired-pulse facilitation ratio, suggesting that changes in presynaptic release do not contribute substantially.

### Dopamine-induced increases in intracellular Ca^2+^

The present study used the Ca^2+^ chelator BAPTA to demonstrate that, similar to findings reported in the prefrontal cortex and the hippocampus [23, 32], the dopamine-mediated facilitation of synaptic currents in the lateral entorhinal cortex is dependent on increases in cytosolic calcium (Fig 1B). Dopamine is unlikely to enhance cytosolic calcium via either T-type voltage-gated calcium channels (VGCCs) which are inhibited by dopamine [68, 69] or via P/Q type channels which are located primarily presynaptically and are typically involved in suppression of synaptic transmission [70]. However, the entorhinal cortex contains both L-type and neuronal N-type voltage-gated calcium channels [46] that can be phosphorylated by PKA [71], and others have also found that D_1_-like receptor activation can enhance calcium influx via L-type and N-type VGCCs [72]. However, we found that blocking either L-type channels with the specific blocker nifidipine, or N-type channels using the L/N type blocker cilnidipine, did not prevent the dopaminergic facilitation of EPSC, indicating that they are not necessary for the dopaminergic facilitation of synaptic transmission.

Cytosolic calcium can also be increased via calcium release from internal sources by activation of InsP_3_ receptors or ryanodine receptors (RyRs). We found that blocking InsP_3_-receptors with heparin blocked the dopaminergic facilitation of synaptic transmission, and that, interestingly, blocking RyRs in a separate group of neurons also fully blocked the dopaminergic facilitation. The dependence of the dopaminergic facilitation on both receptor types suggests that cross-talk between InsP_3_Rs and RyRs may be necessary to amplify the cytosolic calcium concentration via calcium-induced calcium release (CICR). Calcium-induced calcium release can also be triggered by activation of VGCCs, or nicotinic, and metabotropic glutamate receptors [73], and InsP_3_ and RyR receptors may collaborate in propagating CICR in entorhinal neurons. Ryanodine and InsP_3_ receptors are typically segregated in clusters by receptor type, but they remain located proximally to each other [74–76], thus it is possible that calcium release via InsP_3_Rs can activate nearby InsP_3_Rs and RyR channels [77–79]. This may coordinate small, localized calcium signals and amplify them into larger calcium waves via positive feedback, and increase activation of Ca^2+^-dependent kinases (e.g. [80, 81]). Additionally, CICR has also been shown to play a role in mechanisms related to long-lasting synaptic plasticity [82, 83]. In addition, the phosphorylation of InsP_3_ and/or RyR by PKA may contribute to enhancement of release from internal stores, since InsP_3_Rs contain phosphorylation sites for PKA [84–87], which can increase calcium-binding to InsP_3_Rs [88], and potentiate IP_3_-induced calcium flux [83, 89]. Our data suggest that CICR regulated by both InsP_3_Rs and RyR may play a role in the transient regulation of synaptic strength in entorhinal neurons, although further evidence is needed to understand the detailed mechanism of action.

Activation of PI-linked D_1_ receptors which stimulate PLC activity triggers increases in InsP_3_ and calcium release from internal stores [40, 41, 46, 53], and we therefore tested the involvement of this receptor subtype using application of the PI-linked D_1_-receptor agonist SKF83959. SKF83959 lead to a significant increase in EPSCs, and this provides the first evidence for the presence of PI-linked dopamine receptors in the entorhinal cortex. In addition, both the dopaminergic facilitation, and the facilitation induced by SKF83959, was blocked by the PLC inhibitor U-73122 consistent with PI-linked D_1_ receptor activation of PLC and resulting InsP_3_-mediated calcium release. Similar findings in the prefrontal cortex, striatum and hippocampus, show that high levels of PI-linked D_1_ receptor-mediated activation of PLC induce internal Ca^2+^ release [49, 53] and this provides a direct signaling pathway through which dopamine may increase cytosolic calcium levels.

### Calcium-dependent enhancement of AMPA receptor function

Increases in cytosolic calcium can increase the activities of the calcium-dependent protein kinases CaMKII and PKC. Because CaMKII activity is known to modulate glutamatergic synaptic transmission and plasticity [58–60, 24] we examined the effect of the CaMKII blocker KN-93 on the dopamine-induced facilitation of glutamatergic EPSCs. Blocking CaMKII did not block the dopamine-induced facilitation, indicating that CaMKII is not required. In contrast, our findings indicate that inhibition of PKC activity with PKC(19–36) results in a complete block of the dopamine-mediated enhancement of synaptic transmission in lateral entorhinal neurons. Our results that blocking PKC activity blocks the dopamine-induced enhancement of glutamatergic EPSCs are consistent with previously reported data in the nucleus accumbens [90], and with in vitro experiments that have demonstrated that, similar to CaMKII, PKC phosphorylates the AMPA-receptor subunit GluR1 at ser^845^ [58–61]. Activation of PI-linked D_1_-receptors and PLC can enhance PKC via two routes: PLC leads to production of diacylglycerol (DAG) which directly activates PKC, and PLC also increases production of InsP_3_ to enhance cytosolic Ca^2+^ that also activates PKC (Fig 5) [39–41]. Therefore, activation of PI-linked dopamine receptors and PLC may effectively activate PKC through both DAG and Ca^2+^, and this provides a major mechanism through which dopamine may enhance AMPA-mediated synaptic responses in the lateral entorhinal cortex.

### Combined role of PKA- and PKC-dependent signaling

Our work has shown that dopamine leads to a facilitation of synaptic responses in the lateral entorhinal cortex through the combined effect of two major signaling pathways. We have previously shown that the facilitation is dependent on ‘classical’ D_1_-like receptors that stimulate the cAMP-PKA pathway and lead to inhibition of PP1 which is known to dephosphorylate the ser^845^ residue on the AMPA receptor [20]. Inhibition of PP1 can increase EPSCs by promoting the phosphorylation of GluR1 at ser^845^ and enhancing the effects PKA which phosphorylates GluR1 at this same residue [91]. The present results also indicate that the facilitation is dependent on activation of PI-linked D_1_ receptors that increase activity of PLC and PKC. Protein kinase C promotes synaptic transmission by phosphorylating the ser^831^ residue on the GluR1 subunit, and the combined effects of both PKA and PKC on both ser^845^ and ser^831^ residues provides a mechanism for significant changes mediated by increases in both AMPA receptor open-time probability and channel conductance [58–60].

It is not clear why blocking elements in either signaling pathway can fully block the dopaminergic facilitation effect, and we do not currently have data to directly assess possible points of interaction between the pathways. Future studies using protein assays may be useful to determine the precise level of activation and possible sites of interaction between these pathways. The site of interaction must be dependent upon activation of both D_1_ receptor subtypes and be targeted by both signaling pathways. We believe that a probable site for the integration of both signals is adenylyl cyclase. Activation of ‘classical’ G_s/olf_-linked D_1_-receptors enhances adenylyl cyclase and cAMP-PKA activity, which could be further enhanced by activation of PI-linked D_1_ receptors via consequent rises in intracellular calcium. This idea is supported by findings showing that the increases in cytosolic calcium induced by PLC are similar to the concentrations of calcium required to elicit increases in adenylyl cyclase activity in vitro [92]. In this scenario, activation of the PLC pathway alone might induce only partial PKC-dependent phosphorylation of the AMPA GluR1 receptor subunit, but increased Ca^2+^ might allow for enhanced cAMP-PKA-mediated phosphorylation of the AMPA receptor. Although InsP_3_R- and RyR-mediated increases in Ca^2+^ provide a possible mechanism for cooperative interactions between PLC and PKA signaling, it remains unclear why blocking elements of either pathway, such as PKC (Fig 4) or PKA and protein phosphatase 1 [20] could result in an apparent inhibitory effect on activities in the complementary pathway to result in a full block of the facilitation effect.

Of the nine isoforms of adenylyl cyclase, calcium stimulates production of cAMP in isoforms AC1 and AC8. AC1 is found in the entorhinal cortex and throughout the hippocampal formation [93, 94], and similarly, AC8 is expressed abundantly in the entorhinal cortex, piriform cortex and hippocampus [92]. In the hippocampus, mice lacking either isoform show greatly reduced calcium-induced adenylyl cyclase activity, which is linked to impairments in spatial memory and deficits in lasting synaptic plasticity [95–97]. Half-maximal activation of AC1 by cytosolic calcium requires a four-fold lower concentration compared to AC8 (150–200 nM vs 800 nM), thus AC1 may be more sensitive to rises in cytosolic calcium, and subsequent production of cAMP. Although the exact adenylyl cyclase isoform involved in the dopaminergic facilitation remains to be determined, AC1 could be well-suited to act as a “coincidence detector” for the combined activation of G_s_-coupled receptors and increased intracellular calcium because these factors have a synergistic effect on synthesis of cAMP by AC1 that is not displayed by AC8 [98, 99].

## Conclusions

The interplay between the two signaling cascades that we describe here indicates that the dopaminergic facilitation of glutamate-mediated EPSCs in layer II entorhinal neurons does not rely solely on classical D_1_–like receptors and activation of the cAMP-PKA pathway, but that it also depends on PI-linked dopamine receptors that activate a PLC-dependent signaling cascade. The two pathways may interact synergistically via InsP_3_- and calcium-dependent enhancement of cAMP production. Since the dopaminergic facilitation of synaptic transmission described here likely serves to enhance processing of reward-relevant sensory information, the dependence of the facilitation upon the coordinated activity of two signaling cascades may serve to gate or limit increases in synaptic transmission to instances of intense and/or prolonged release of dopamine that could provide sufficient activation of both pathways. Further, it is also possible that the complexity of the signaling mechanisms that mediate the dopaminergic facilitation of synaptic transmission may allow for multiple points in the signaling process that may be modulated by activation of other neuromodulatory transmitter receptors as observed in other brain regions [53, 100–102].

## Supporting Information

S1 ARRIVE Checklist ARRIVE Ethics Checklist. (PDF)
